# Effects of Human Activities on the Spatial Distribution, Ecological Risk and Sources of PTEs in Coastal Sediments

**DOI:** 10.3390/ijerph182312476

**Published:** 2021-11-26

**Authors:** Weili Wang, Cai Lin, Lingqing Wang, Ronggen Jiang, Yang Liu, Hui Lin, Jinmin Chen

**Affiliations:** 1 Third Institute of Oceanography, Ministry of Natural Resources, Xiamen 361005, China; lincai@tio.org.cn (C.L.); jiangronggen@tio.org.cn (R.J.); jczx_liuyang@tio.org.cn (Y.L.); linhui@tio.org.cn (H.L.); chenjinmin@tio.org.cn (J.C.); 2 Institute of Geographical Sciences and Natural Resources Research, Chinese Academy of Sciences, Beijing 100101, China; wanglq@igsnrr.ac.cn

**Keywords:** potentially toxic elements, Xiamen Bay, sediment, PMF model, risk assessment

## Abstract

Potentially toxic elements (PTEs) have attracted substantial attention because of their widespread sources, long residue time and easy accumulation. PTEs in the surface sediments of inshore waters are strongly affected by human activities because these waters are a zone of interaction between the ocean and land. In the present study, to explore the environmental geochemical behaviour and source of PTEs in the surface sediments of coastal waters, the contents and spatial distributions of copper (Cu), lead (Pb), zinc (Zn), cadmium (Cd), chromium (Cr), mercury (Hg) and arsenic (As) in different regions of Xiamen Bay were investigated. The data were processed by multivariate statistical methods, and the distribution characteristics of PTEs in the surface sediments of Xiamen Bay were analysed. In addition, the pollution load index (PLI), geo-accumulation index (Igeo) and potential ecological index(RI) were used to evaluate the pollution degree and potential risk in the surface sediments of Xiamen Bay, and the positive matrix factorisation (PMF) model was used to analyse the source. The results show that Zn had the highest mean concentration, followed by Pb, Cr, Cu, As, Cd and Hg, among the seven PTEs. The mean contents of Pb, Zn, Cd, Cu and Hg, and especially Hg and Cd, were higher than the corresponding environmental background values. The average PLI value indicated that the Xiamen Bay sediment was moderately contaminated by PTEs. The Igeo results showed that Xiamen Bay was moderately to strongly polluted by Cd and Hg. The proportions of samples with low, medium and strong risk levels were 11.63%, 74.42%, and 13.95% in surface sediments, respectively. PMF models showed that the input of chemical fertilizer and medication, anthropogenic atmospheric components and terrestrial detritus were the main sources of PTEs in the surface sediment of Xiamen Bay.

## 1. Introduction

As an important part of the marine ecosystem, sediment is the main enrichment medium for terrestrial pollutants after they enter the sea and an important habitat for aquatic organisms [1]. Due to its higher stability than that of water medium, sediment can more accurately indicate the quality status and trend of the marine environment [2,3,4]. Potentially toxic elements (PTEs) are important pollutants of concern in the marine environment and mainly originate from surface runoff, atmospheric deposition and direct offshore pollution [5,6,7,8]. They can be deposited in sediments through the adsorption of suspended particulates [5,9,10]. Due to the characteristics of accumulation and long residue time, PTEs easily accumulate in benthic organisms and transfer to predators or human bodies through the food chain [11], thereby endangering aquatic organisms and human health [7,12,13,14]. When the conditions external to the sediment (such as pH and redox conditions) change, the accumulated PTEs in the sediment may be released into the water again [15]. Therefore, PTEs in sediments not only have a direct toxic effect on benthic organisms but also pose potential threats to the ecosystem through secondary release, bioaccumulation, and amplification [16].

Previous studies have found that the concentration and distribution of PTEs in marine sediments are mainly affected by particle size, natural sources and human activities [17,18,19]. Most of the PTEs were transported to the ocean in the form of weathered debris. In addition, wastewater containing PTEs closely related to human activities had also been discharged into the sea, and all these PTEs entering the sea accumulated in sediments, resulting in PTEs pollution [20,21,22].

Xiamen Bay, which is located in the western Taiwan Strait of China, is an important sea outlet along the southeastern coast of China. There are many islands and reefs in the sea area, with vertical and horizontal ditches and branches, and they are filled by the Jiulong River and Xi River. The sedimentary pattern, environment and process of sediments in Xiamen Bay are significantly affected by terrestrial sources. There are many types of sediments in Xiamen Bay, mainly clayey silt [23]. The clay minerals in the sediments are mainly illite, kaolinite, chlorite and montmorillonite, and Jiulong River runoff transport is the main factor for the formation of clay mineral deposits in Xiamen Bay. The rapid development of the regional economy has also brought much pollution to coastal waters [24,25]. In the present study, the contents of PTEs in the surface sediments of Xiamen Bay (Jiulong River Estuary, Xiamen West Harbour, Tongan Bay, Eastern Channel and Southern Channel) were investigated, and our aims were to (1) analyse the spatial differences and factors influencing the distribution of PTEs in sediments; (2) evaluate the pollution degree and potential biological hazards of PTEs; and (3) explore the main source of PTE pollution in surface sediments, which helps to correctly delineate the functions of different sea areas, reasonably protect the ocean and ensure the sustainable development of the marine economy in Xiamen Bay.

## 2. Materials and Methods

### 2.1. Study Area

**Figure 1:**
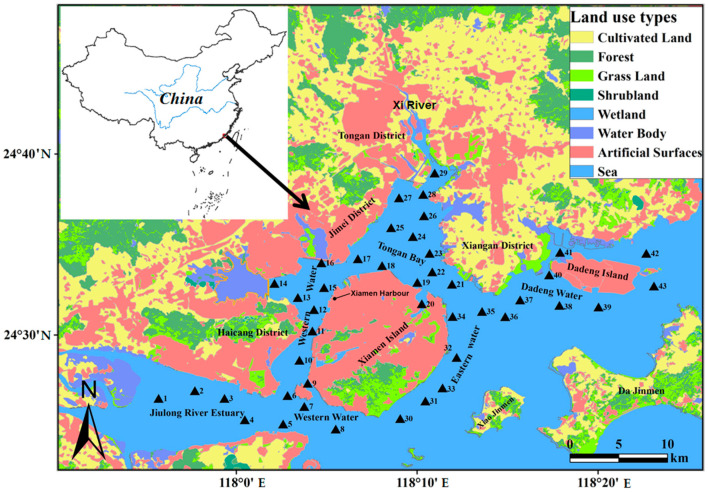
Sampling sites for surface sediments in Xiamen Bay (▲: Sediment Sampling; 1–43: Station numbers).

Xiamen Bay is a semi-closed compound estuarine bay in southern Fujian Province, China [26]. The water area is 154.18 km^2^, with a depth of 10 m extending along the centre of the outer and inner harbours, and the maximum water depth is 31 m. The Jiulong River, the largest river in Xiamen Bay, has an annual maximum runoff of 288 × 10^8^ m^3^ [27,28]. Xiamen Bay is located in the economically developed coastal area of China, which have frequent human activities [29,30]. In order to better study the content and distribution characteristics of PTEs in Xiamen Bay sediments, according to the ecological environment, industrial layout and surrounding administrative divisions, the study area was divided into 6 sub-regions: Jiulong River Estuary (sites 01~04), Southern Waters (sites 05~08), Western Waters (sites 09~16), Tongan Bay (sites 17~29), Eastern Waters (sites 30~35) and Dadeng Waters (sites 36~43) (Figure 1).

### 2.2. Sampling and Analysis

A total of 43 surficial sediment samples were collected at depths approximately between 0 and 5 cm with a grab-type dredger. We used the hydrological winch to settle the clam mud collector to the selected sampling point for sampling. When sampling, we opened the upper cover of the mud collector and gently tilt the mud collector to make the upper water flow out slowly. According to the specification for marine monitoring (GB17378.3-2007), in order to make the sediment samples representative, we sampled 2–3 times around the same sampling point and mixed the collected samples evenly. These samples were collected with clam grab and the undisturbed part of the surface layer was placed in a polyethylene sealed bag that was soaked in a dilute nitric acid solution, dried, refrigerated, and brought back to the laboratory in 8 to 10 May 2017 (Figure 1).

After air drying, the samples were ground and sieved for further pretreatment.

PTEs in the sediments were tested and analysed by different methods. Among them, Cu, Zn, Cr, Cd, As and Pb were digested (0.5 g) by the HNO_3_-HF-HClO_4_ system and detected by a 7700X ICP-MS produced by Agilent (Santa Clara, CA, USA). The level of Hg was detected by an AFS-930 atomic fluorescence spectrometer by Titan Instruments (Beijing, China).

The content of sulfide in sediments was determined by methylene blue spectrophotometry, and the concentration of total organic carbon (TOC) in sediments was determined by the potassium dichromate redox volumetric method.

All samples to be processed were measured in parallel at 10% of the total amount to check possible errors during sample processing. A blank sample test was used to eliminate systematic errors. The internal standard method was used to test and compensate for the fluctuation in the analytical signal to improve the precision and accuracy of determination. The recovery rate of the standard addition was tested by adding the same sample to every 10–20 samples. The recovery rate of the reference materials was 85~115%, meeting the quantification limits (80–115%).

### 2.3. Data Processing

To analyse the source characteristics of PTEs and determine the characteristics affecting the content and geochemical distribution of PTEs in sediments, Pearson correlation analysis was carried out for 7 kinds of PTEs in sediments and their geochemical parameters, such as TOC. SPSS 20.0 was used for data processing and correlation analysis. The spatial distribution of PTEs was made by inverse distance interpolation through ArcGIS 10.3. In the above statistical analysis, the significant difference level was expressed as *p* < 0.05.

### 2.4. Sediment Contamination and Risk Assessment Methods

#### 2.4.1. Pollution Load Index (PLI)

The pollution load index (PLI) is widely used to determine the pollution status of all PTEs in studies [16,31,32,33]. Before using the pollution load index (PLI) for evaluation, the contamination factors (CFs) of each metal element need to be calculated. As a simple and rapid method, CFs have been widely used to calculate the pollution degree of a single metal element [34,35,36]. The CFs and PLI of PTEs in surface sediments were calculated using Equations (1) and (2), respectively:

(1) $$\mathrm{CF}=\frac{C_{i}}{C_{n}}$$

(2) $$\mathrm{PLI}=\sqrt[{n}]{{\mathrm{CF}}_{1}{\;\times \;\mathrm{CF}}_{2}\cdots {\mathrm{CF}}_{n}}$$

where ${\;C}_{i}$ represents the monitored concentration of the element metal i and $C_{n}$ represents the corresponding sediment background value (Table 1. Background Value). According to previous studies, the CF can be classified into four contamination levels [36,37]: low pollution (<1); moderate pollution (1~3); considerable pollution (3~6); and very high pollution (≥6). The PLI can be classified into two cases according to the PLI value of total PTEs: no contamination (PLI ≤ 1) and contamination (PLI > 1) [15].

#### 2.4.2. Geo-Accumulation Index (Igeo)

The geo-accumulation index (Igeo), which was first proposed by Muller [37], is a quantitative index to study PTEs pollution in sediments. It is widely used to study the contamination of PTEs pollution in modern sediments [38,39,40]. To characterise the level of contamination within the sediment, Igeo values were measured using the formula given below:

(3) $$I_{\mathrm{geo}}{=\log}_{2}{(C}_{x}/1{.5B}_{x})$$

where C_x_ is the measured concentration of metal element x in the samples; B_x_ is the reference or background value of element x (Table 1. Background Value).

According to the Igeo value, the pollution degree of PTEs in sediments can be divided into 7 levels [41,42]:

(4) $$\mathrm{Igeo}=\{\begin{array}{c} \;<0\;\;\;\;\;\;\;\;\;\;\;\;\;\;\;\;\;\;\;\;\;\;\;\;\;\;\;\mathrm{no}\;\mathrm{pollution}\\ \;0\sim 1\;\;\;\;\;\;\;\;\;\;\;\;\mathrm{no}\;\mathrm{to}\;\mathrm{moderate}\;\mathrm{pollution}\\ 1\sim 2\;\;\;\;\;\;\;\;\;\;\;\;\;\mathrm{moderate}\;\mathrm{pollution}\\ \;\;\;\;\;\;2\sim 3\;\;\mathrm{moderate}\;\mathrm{to}\;\mathrm{strong}\;\mathrm{pollution}\\ 3\sim 4\;\;\;\;\;\;\;\;\;\;\;\;\;\;\;\mathrm{strong}\;\mathrm{pollution}\\ \;\;\;\;\;4\sim 5\;\;\mathrm{strong}\;\mathrm{to}\;\mathrm{extreme}\;\mathrm{pollution}\\ >5\;\;\;\;\;\;\;\;\;\;\;\;\;\;\;\mathrm{extreme}\;\mathrm{pollution} \end{array}$$

#### 2.4.3. Ecological Risk Assessment

In this study, the potential ecological index (RI) from Swedish scientist Hakanson [43] was cited to assess the ecological risk in the surface sediments of Xiamen Bay. This method obtains the pollution coefficient of PTEs through the single factor index method, combined with the toxicity coefficient of different metal elements, to evaluate the degree of harm of PTEs quickly and easily to the ecological environment [44,45]. The RI was calculated using Equations (5) and (6) [6]:

(5) $$E_{r}^{i}{=T}_{r}^{i}\;\times \;\frac{C^{i}}{C_{n}^{i}}$$

(6) $$\mathrm{RI}=\overset{m}{\underset{i=1}{\sum }}E_{r}^{i}$$

where C^i^ is the content of metal i in the sediment; C^i^_n_ is the background content of metal i in the sediment (Table 1. Background Value); E^i^_r_ is the potential ecological risk coefficient of element i; and T^i^_r_ is the toxicity response factor of each element (T^Cu^_r_ = 5; T^Pb^_r_ = 5; T^Cd^_r_ = 30; T^Zn^_r_ = 1; T^Cr^_r_ = 40; T^As^_r_ = 1; T^Hg^_r_ = 10).

The categories for E^i^_r_ and RI were as follows in Equations (7) and (8), respectively.

(7) $$E_{r}^{i}=\{\begin{array}{c} \;<40\;\;\;\;\;\;\;\;\;\;\;\;\;\;\;\;\;\mathrm{Low}\;\mathrm{risk}\;\mathrm{degree}\\ \;40–80\;\;\;\;\;\;\;\;\;\mathrm{Moderate}\;\mathrm{risk}\;\mathrm{degree}\\ 80–160\;\;\;\;\;\;\;\mathrm{Considerable}\;\mathrm{risk}\;\mathrm{degree}\\ 160–320\;\;\;\;\;\;\;\;\;\;\;\mathrm{Very}\;\mathrm{high}\;\mathrm{risk}\;\mathrm{degree}\\ \;>320\;\;\;\;\;\;\;\;\mathrm{Disastrous}\;\mathrm{risk}\;\mathrm{degree} \end{array}$$

(8) $$\mathrm{RI}=\{\begin{array}{c} \;<150\;\;\;\;\;\;\;\;\;\;\;\;\;\;\;\;\;\mathrm{Low}\;\mathrm{risk}\;\mathrm{degree}\\ \;150–300\;\;\;\;\;\;\;\;\;\;\mathrm{Moderate}\;\mathrm{risk}\;\mathrm{degree}\\ \;300–600\;\;\;\;\;\;\mathrm{Considerable}\;\mathrm{risk}\;\mathrm{degree}\\ >600\;\;\;\;\;\;\;\;\;\;\;\;\;\;\;\;\;\;\;\;\;\;\;\;\;\mathrm{High}\;\mathrm{risk}\;\mathrm{degree} \end{array}$$

#### 2.4.4. Positive Matrix Factorisation (PMF)

A positive matrix factorisation (PMF) model can not only optimize data through the standard deviation of the data but also deal with missing and inaccurate data to ensure data reliability [40,46]. This method is a quantitative method for the matrix analysis of pollution sources based on the receptor model. It composes a matrix of collected samples and a variety of PTEs, which is decomposed into the contribution rate matrix of the pollution source and the pollution source component spectrum matrix through matrix operation, and then iterative operation is carried out through the least squares method to obtain the pollution source composition [46,47].

**Table 1:** Descriptive statistics for PTEs in surface sediment in Xiamen Bay and other areas.

PTEs (mg/kg)	Cu	Pb	Zn	Cd	Cr	Hg	As	References
Min	0.4	12.3	16.3	0.032	1.5	0.006	1.9	
Max	69.6	68.2	188.0	0.419	70.0	0.160	13.1	
Average	18.8 ± 13.7	41.4 ± 14.8	82.5 ± 32.6	0.135 ± 0.084	20.6 ± 15.1	0.079 ± 0.033	7.0 ± 2.6	
Coefficient of Variation	73.1%	35.7%	39.5%	62.6%	73.3%	41.7%	36.7%	
Kurtosis	−1.14	5.21	4.39	−0.85	1.41	3.64	2.76	
Skewness	0.15	2.08	1.94	−0.14	0.66	1.76	1.67	
Bohai Bay, China	32.6 ± 7.0	26.9 ± 3.4	95.2 ± 19.7	0.300 ± 0.1	75.2 ± 13.1	0.072 ± 0.042	12.9 ± 3.1	[48]
Hangzhou Bay, China	27.2 ± 6.0	21.2 ± 17.3	86.0 ± 17.3	0.214 ± 0.070	61.7 ± 10.3	0.050 ± 0.011	9.4 ± 5.0	[49]
Daya Bay, China	16.5 ± 6.3	37.0 ± 8.5	87.8 ± 26.4	0.07 ± 0.02	59.0 ± 16.6	0.04 ± 0.01	8.16 ± 1.99	[50]
Thessaloniki Bay, Greece	77.2 ± 46.5	84.2 ± 53.8	218 ± 157.9	2.51 ± 3.57	115.4 ± 34	-	-	[51]
Class I, China	35.0	60.0	150.0	0.50	80.0	0.20	20.0	[52]
Class II, China	100.0	130.0	350.0	1.50	150.0	0.50	65.0	[52]
Background Value	15.0	20.0	65.0	0.065	60.0	0.025	7.7	[53]

## 3. Results and Discussion

### 3.1. Geochemistry and Distribution of PTEs

Table S1 shows the concentrations of PTEs in each sampling station in Xiamen Bay and the Table 1 shows the descriptive statistical analysis and the background values of the PTEs contents in the surface sediments in Xiamen Bay. The average abundance data of elements in the earth’s crust or the average shale value are often used as its environmental background value, while Xu et al. [52] believe that the regional background value may be more appropriate. In this study, the baseline/background metal concentrations in continental shelf sediments of China were used as the background metal values shown in Table 1 [53]. Among the seven PTEs in the investigated Xiamen Bay sediments, Zn had the highest mean content, followed by Pb, Cr, Cu, As, Cd and Hg, with contents of 82.5 ± 32.6 mg/kg, 41.4 ± 14.8 mg/kg, 20.6 ± 15.1 mg/kg, 18.8 ± 13.7 mg/kg, 7.0 ± 2.6 mg/kg, 0.135 ± 0.084 mg/kg and 0.079 ± 0.033 mg/kg, respectively. The coefficients of variation of Cu, Cd and Cr were higher than those of Hg, Zn, Pb and As, indicating that Cu, Cd and Cr in sediments had a large spatial diversity.

According to Table 1, the average contents of copper, lead, zinc, cadmium and mercury were higher than the sediment background values, especially those of mercury and cadmium, which were more than two times the background values. Referring to the marine sediment quality in China (GB18668-2002) [52], the average contents of the seven PTEs in surficial sediments were lower than the limit of the class I sediment quality standard. The average contents of PTEs in Xiamen Bay were similar to those in other coastal regions in China and much lower than those in Thessaloniki Bay, Greece. A previous study found that the accumulation of PTEs in sediments was related to the frequency of economic activities [54].

**Figure 2:**
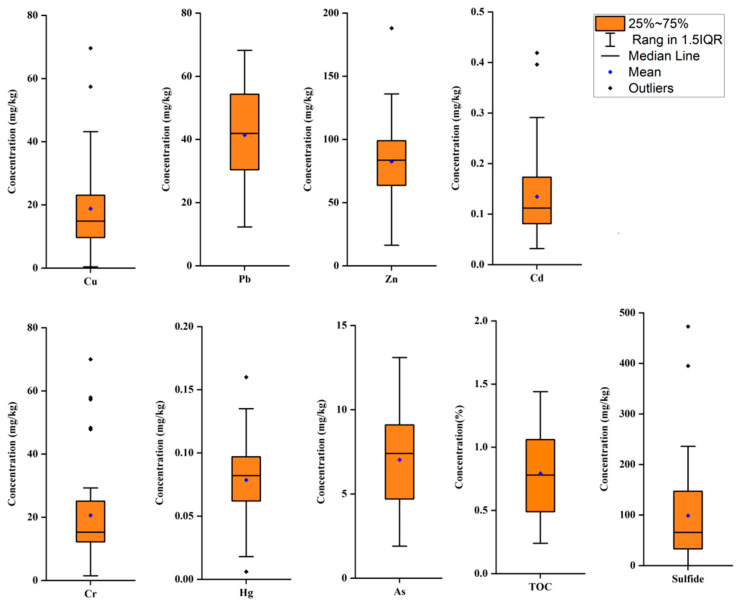
Box diagram showing the average contents of PTEs, TOC and Sulfide in Xiamen Bay.

**Figure 3:**
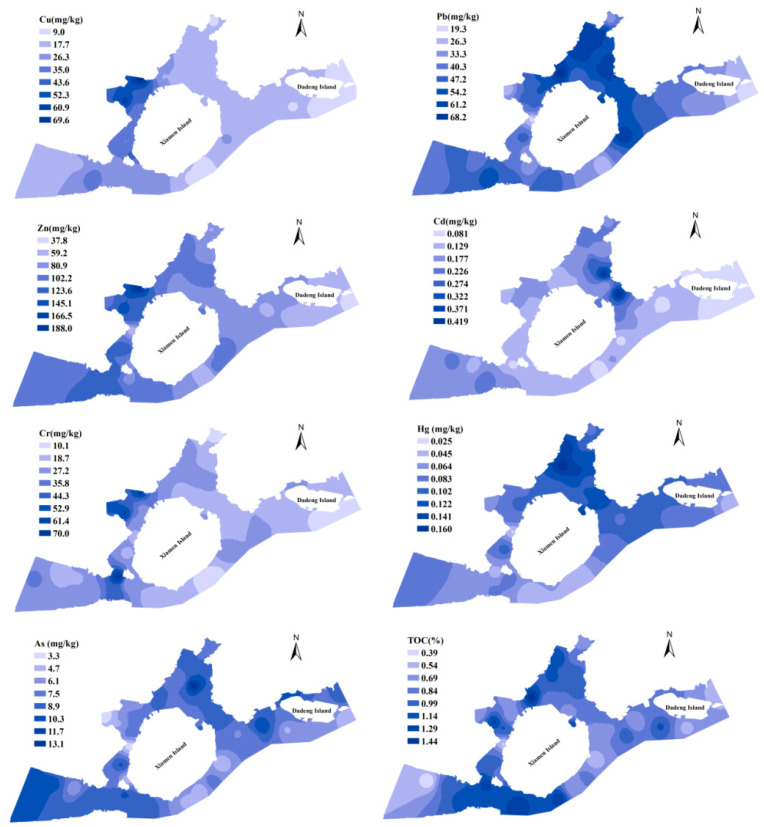
Spatial distribution of the PTEs contents (including Cu, Pb, Zn, Cd, As, Cr and Hg) and TOC in the surface sediments of Xiamen Bay.

The average contents of PTEs in surface sediment from Xiamen Bay are shown in Figure 2, and the spatial interpolation graph is presented in Figure 3. The average contents of copper and zinc in different areas were as follows: Western Waters > Southern Waters > Jiulong River Estuary > Tongan Bay > Eastern Waters > Dadeng Waters; the mean concentrations of Cr were as follows: Southern Waters > Western Waters > Jiulong River Estuary > Tongan Bay > Eastern Waters > Dadeng Waters. The contents of Cu, Cr and Zn had similar spatial distributions (Figure 3); the Southern Waters and Western Waters were the highest, and the Dadeng Waters were the lowest. The mean contents of lead, mercury and arsenic were higher in the Tongan Bay, Jiulong River Estuary and Western Waters and lower in the Dadeng Waters and Southern Waters. The average contents of Cd in surficial sediments were in descending order: Tongan Bay > Jiulong River Estuary > Western Waters > Eastern Waters > Southern Waters > Dadeng Waters.

The average concentrations of sulfide and TOC in the surface sediments were 98.8 ± 98.5 mg/kg and 0.79 ± 0.32%, with coefficients of variation (CVs) of 99.7% and 40.3%, respectively. The mean concentrations of sulfide and TOC were lower than the class I standard of marine sediment quality.

### 3.2. Pollution Index Evaluation

#### 3.2.1. CFs and PLI

**Figure 4:**
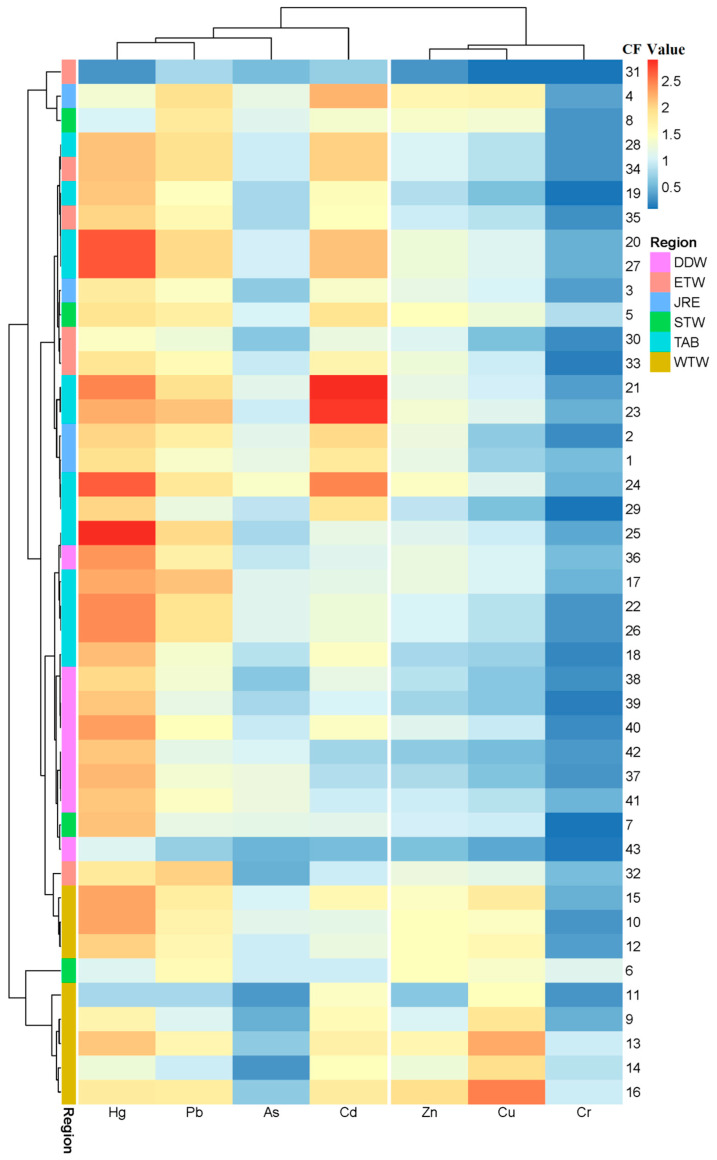
Cluster heatmap of the contamination factor (CF).

The contamination factor (CF) was deemed a useful method to evaluate the accumulation of PTEs in surface sediments over time [53]. The cluster heatmap of CFs from all sampling sites is presented in Figure 4. The CF ranges of copper, lead, zinc, cadmium, chromium, arsenic and mercury in the surface sediments of Xiamen Bay were 0.03 to 4.64, 0.62 to 3.41, 0.25 to 2.89, 0.49 to 6.45, 2.07 to 0.03, 0.25 to 1.70, 0.24 to 6.40, and 0.19 to 1.96, with average CFs of 1.25, 2.07, 1.27, 2.07, 0.34, 0.91, 3.14 and 1.21, respectively (Table 2, Figure 4). Among the different research areas, the Western Waters had the highest average CFs for Cu and Zn, and Tongan Bay had the highest mean CFs of Pb, Cd and Zn. The highest average CFs for Cr (0.6) and As (1.1) were all in the Southern Waters. The CF results showed that Xiamen Bay had low contamination levels and was moderately polluted by Cu, Pb, Zn, Cd, Cr and As, and it had low pollution to very high contamination levels of Hg. Hg had a maximum CF at site D25 in Tongan Bay, indicating that these sites were highly contaminated, and 27 of the 43 stations were considerably polluted by Hg.

**Figure 5:**
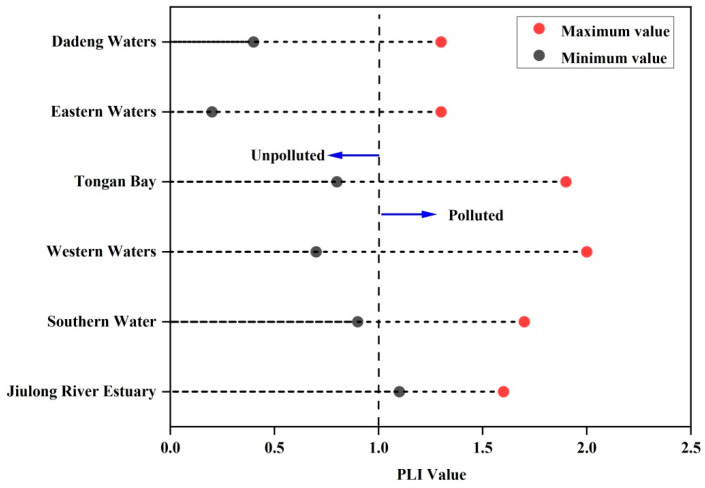
Lollipop chart of the pollution load index (PLI) in the subregions of Xiamen Bay.

The pollution load index (PLI) has been adopted by many researchers to explain the overall impact of PTEs pollution [40]. The PLI value (>1) indicated that the surface sediment quality was polluted [55]. The PLI was between 0.2 and 2.0 in Xiamen Bay sediments (Figure 5). The average PLI values followed the order of the following regions: Western Waters > Tongan Bay > Jiulong River Estuary > Southern Waters > Dadeng Waters > Eastern Waters. There were four areas with PLIs > 1 in the five study areas, indicating that these four regions were contaminated by PTEs. According to the PLI classification, most of the sediment sites were contaminated (PLI > 1). However, higher PLI values indicate that Hg and Cd were the leading causes of sediment pollution. The average PLI value indicated that the Xiamen Bay sediment was moderately polluted by PTEs.

#### 3.2.2. Igeo Results

**Figure 6:**
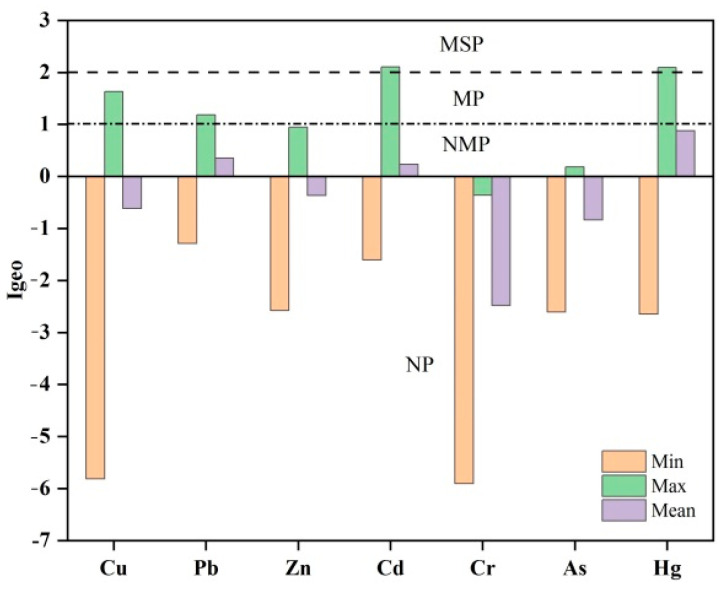
Histogram of the Igeo in the surface sediment of Xiamen Bay (NP = no pollution; NMP = no to moderate pollution; MP = moderate pollution; MSP = moderate to strong pollution).

Igeo is a method to divide the PTE sediment contents into different pollution levels by multiplying its background value. According to Figure 6, the Igeo range of each PTE was −5.81~1.63 for Cu, −1.29~1.18 for Pb, −2.58~0.95 for Zn, −1.61~2.10 for Cd, −5.91~−0.36 for Cr, −2.60~0.18 for As, and −2.64~2.09 for Hg, with average values of −0.62 (Cu), 0.35 (Pb), −0.36 (Zn), 0.23 (Cd), −2.48 (Cr), As (−0.84) and Hg (0.88), respectively. The Igeo values indicated that Cr did not contaminate the Xiamen Bay sediment (Igeo < 1) and the sediment was unpolluted to moderately polluted by Zn and As, moderately contaminated by Cu and Pb (Igeo < 2), and moderately to strongly contaminated by Cd and Hg.

#### 3.2.3. Ecological Risk Assessment

**Figure 7:**
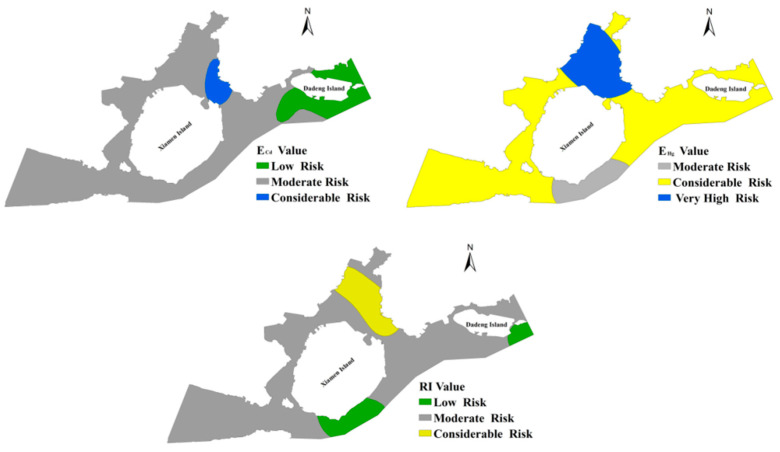
Spatial distribution map of the potential ecological risk of PTEs in Xiamen Bay.

The Hakanson ecological risk index (RI) not only considers the concentration of different metal elements but also considers the biological toxicity response of different PTEs and is one of the most commonly used potential ecological hazard assessment methods [43]. The potential ecological hazards of seven PTEs in the sediments of Xiamen Bay are in order from strong to weak: Hg (125.7) > Cd (62.1) > Cr (13.7) > Pb (10.3) > As (9.1) > Cu (6.3) > Zn (1.3). The ecological risk indices of Zn, As, Cu and Pb were all less than 40, which were at the level of slight ecological risk and had little impact on the potential ecological risk of surface sediments in Xiamen Bay. Cr at one sampling site reached the level of moderate ecological risk, and all other sample points were at the level of slight ecological risk, indicating that chromium made little contribution to the ecological risk of sediment in Xiamen Bay. The samples of Cd at slight ecological risk levels accounted for 32.56% of the total samples, the percentage of samples at medium ecological risk levels was 44.18%, and the percentages of samples at strong ecological risk levels reached 18.61% and 4.65%, respectively. A total of 32.45% of the Hg samples reached the medium ecological risk level, 64.9% of the samples reached the strong ecological risk level, and 1.53% and 0.18% of the samples reached the strong and extremely strong ecological risk levels, respectively. The potential ecological risks of PTEs in the surface sediment in Xiamen Bay were mainly caused by Cd and Hg (Figure 7). Previous studies found that Hg and Cd had higher hazard risks for sediments along coastal regions in China [6,48,56,57].

The RI values of surface sediments in Xiamen Bay that were calculated based on the PTEs content of 43 sampling points were between 38.4 and 418.6, with an average value of 228.5, indicating that the surface sediments in Xiamen Bay had moderate potential ecological hazard according to the risk level. As shown in Figure 7, the proportions of samples with low, medium and strong risk levels were 11.63%, 74.42% and 13.95%, respectively, and most of the samples were at a medium risk level. The average RI values of sediment in the studied regions were in the following order: Tongan Bay > Jiulong River Estuary > Western Waters > Dadeng Waters > Southern Waters > Eastern Waters. The highest RI value was observed at Tongan Bay.

### 3.3. Source Analysis of PTE Pollution in Sediments

**Figure 8:**
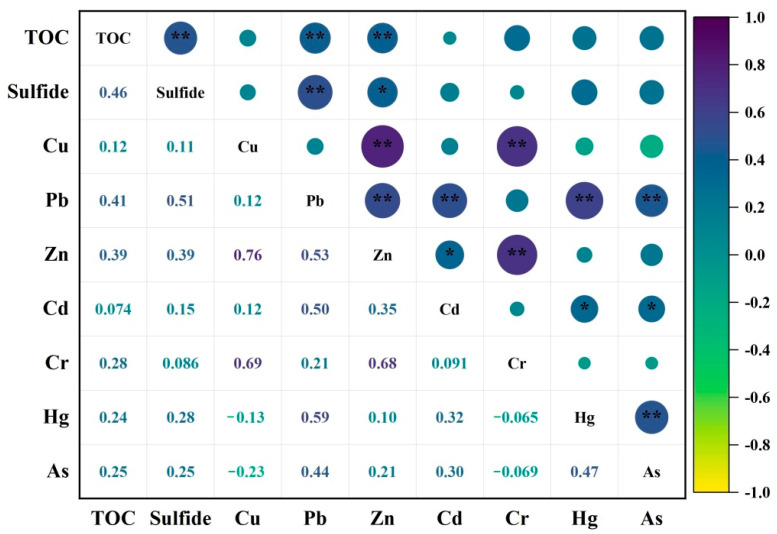
Correlation analysis of PTEs, TOC and sulfide in the surface sediment of Xiamen Bay (* *p* ≤0.05, ** *p* ≤ 0.01.).

The Pearson correlation analysis of PTEs in sediments in Xiamen Bay is shown in Figure 8. Sulfide and TOC had significant correlations with lead and zinc but not with other PTEs, while the correlation coefficient between sulfide and organic carbon was high. Previous studies have reported that organic matter in sediments plays a key role in the content distribution, migration and transformation of PTEs, and there is a significant correlation between them [58,59], while other studies have found that there is no correlation between some metals and organic carbon in sediments, which may be due to the circulation and redistribution of metal elements caused by human input and natural depositional processes [60,61]. There were significant positive correlations between Cu, Zn and Cr in the surface sediment, indicating that they may have similar or the same sources. Previous studies have shown that Cr is less affected by anthropogenic influence and is mainly controlled by the parent rock [19,62]. According to Figure 8., there were high positive correlations between lead and zinc, cadmium, mercury and arsenic, which suggests that these five metals originated from similar pollution sources and methods.

**Figure 9:**
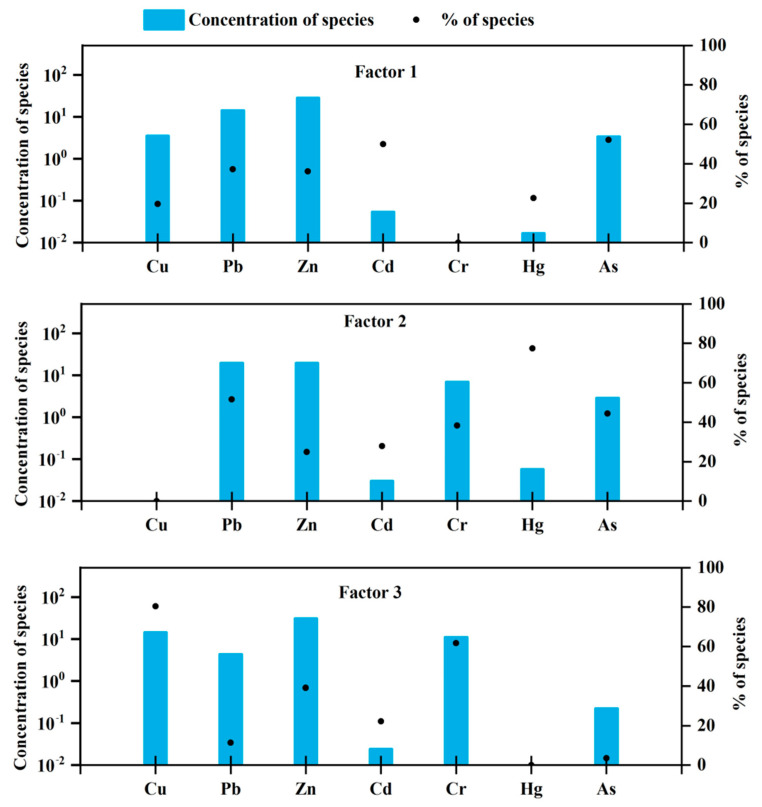
Source profile ratios in the sediments of Xiamen Bay for different PTEs.

In the present study, positive matrix factorisation (PMF) was used to further explore the sources of PTEs in the surface sediment of Xiamen Bay. The trend of the source composition spectrum and contribution rate of three factors of PMF source analysis is shown in Figure 9. Factor 1 had the highest contribution rate to Cd and As and the lowest contribution rate to the total amount and concentration of Cr. Previous studies have shown that As and Cd are generally considered to be related to the use of chemical fertilisers and pesticides in human agricultural activities. Factor 1 was the input of chemical fertiliser and medication from human activities.

Factor 2 was characterised by high mercury and lead contents. Some previous studies have found that Pb in marine sediments mainly comes from natural sources, such as volcanic eruptions and forest fires, as well as atmospheric deposition produced by human activities [53,63]. Research has shown that mercury in sediments comes from the use of fossil fuels, such as coal-fired plants [36]. Therefore, factor 2 was mainly anthropogenic atmospheric input.

The elements with higher load ratios in factor 3 included Cu and Cr, with contribution rates of 80.39% and 61.73%, respectively. The average values of Cr and Cu in the sediments did not exceed the elemental background values, and the pollution evaluation grades were clean. Therefore, factor 3 was mainly the input of terrestrial detritus.

## 4. Conclusions

In this study, the concentration and spatial distribution of PTEs in surface sediments in different areas of Xiamen Bay were comprehensively analysed. We evaluated the pollution degree and potential ecological risk of PTEs through the PLI, Igeo and RI and used the PMF model to explore their possible anthropogenic sources. The results of the study showed that there were significant differences in the contents and distribution of the seven selected PTEs in different sea areas of Xiamen Bay. Except for Cr and Zn, the PTEs had different levels of ecological risks; Cd and Hg had higher ecological risks. The results of the PMF model showed that natural sources, anthropogenic atmospheric inputs, and chemical fertilizers and drugs were the main sources of PTE pollution in the surface sediments of Xiamen Bay. In particular, the Hg content was significantly affected by human activities, which should be given special attention.

## Figures and Tables

**Table 2:** The contamination factor (CF) and pollution load index (PLI) of the PTEs in the surface sediments of Xiamen Bay.

PTEs Pollution Index		Cu	Pb	Zn	Cd	Cr	As	Hg	PLI
**CF**	Min.	0.03	0.62	0.25	0.49	0.03	0.25	0.24	0.19
	Max.	4.64	3.41	2.89	6.45	1.17	1.70	6.40	1.96
	Mean	1.31	2.06	1.23	2.13	0.34	0.87	3.33	1.19

## Data Availability

The data presented in this study are available on request from the corresponding author.

## Supplementary Materials

The following are available online at https://www.mdpi.com/article/10.3390/ijerph182312476/s1, Table S1: Each sampling station in Xiamen Bay.
