# Supplementary material for: Effects of Human Activities on the Spatial Distribution, Ecological Risk and Sources of PTEs in Coastal Sediments
Source: Int J Environ Res Public Health. 2021 Nov 26;18(23):12476. doi: 10.3390/ijerph182312476 (PMC8657197; doi:10.3390/ijerph182312476)
Supplement: Supplementary file 1 [file ijerph-18-12476-s001.zip › ijerph-1414106-supplementary.pdf]

Table S1. Each sampling station in Xiamen Bay.

Station	TOC (%)	Sulfide (mg/kg)	Cu (mg/kg)	Pb (mg/kg)	Zn (mg/kg)	Cd (mg/kg)	Cr (mg/kg)	Hg (mg/kg)	As (mg/kg)
1	0.45	17.2	10.0	32.3	83.6	0.164	29.0	0.070	10.2
2	0.24	48.3	8.9	47.2	91.1	0.194	12.2	0.077	9.5
3	0.94	222.0	15.7	34.3	85.2	0.105	17.0	0.062	4.7
4	1.06	473.0	31.8	55.8	135.0	0.234	19.3	0.039	10.0
5	1.20	140.0	21.3	46.2	120.0	0.176	47.8	0.069	8.1
6	1.24	69.8	24.6	39.7	119.0	0.063	70.0	0.028	7.5
7	1.13	68.2	14.9	25.9	64.8	0.080	5.4	0.084	9.6
8	1.27	87.0	23.1	51.0	105.0	0.104	13.6	0.026	9.2
9	0.65	30.2	40.1	22.5	71.4	0.127	23.4	0.053	3.0
10	0.90	216.0	26.0	44.5	119.0	0.083	13.7	0.098	9.4
11	0.32	11.8	28.0	14.6	36.4	0.113	13.6	0.018	2.0
12	1.03	156.0	30.4	42.0	121.0	0.087	17.4	0.078	7.4
13	1.23	56.3	57.4	41.9	136.0	0.148	57.9	0.082	4.5
14	0.46	33.1	43.2	19.7	93.5	0.120	48.3	0.036	1.9
15	0.50	39.3	37.4	48.6	113.0	0.130	23.2	0.097	8.2
16	0.51	64.9	69.6	47.7	188.0	0.160	57.3	0.062	4.6
17	1.44	147.0	16.7	68.2	88.5	0.081	25.6	0.095	9.1
18	0.61	34.8	9.7	31.5	47.1	0.112	9.8	0.087	6.2
19	0.42	52.6	7.7	36.0	51.3	0.124	4.1	0.082	5.6
20	1.20	101.0	16.9	59.4	92.0	0.218	23.4	0.135	7.7
21	0.78	65.7	15.3	56.2	85.8	0.419	17.6	0.113	9.4
22	0.78	200.0	12.4	54.3	68.0	0.095	14.4	0.110	8.9
23	0.94	113.0	17.9	66.9	99.0	0.396	22.5	0.093	7.4
24	0.85	86.8	17.9	52.0	112.0	0.291	25.1	0.133	13.1
25	1.07	176.0	14.7	60.0	77.1	0.085	20.5	0.160	5.6
26	0.86	155.0	12.4	54.3	68.0	0.095	14.4	0.110	8.9
27	1.20	395.0	16.9	59.4	92.0	0.218	23.4	0.135	7.7
28	0.73	0.0	12.5	56.3	71.6	0.203	14.4	0.085	7.4
29	0.57	82.8	7.6	27.2	57.5	0.173	3.8	0.077	6.7
30	1.25	27.9	7.7	28.4	74.3	0.088	11.2	0.043	4.4
31	0.35	0.0	0.4	14.7	16.3	0.041	1.5	0.006	3.7
32	0.39	168.0	19.2	62.3	91.1	0.064	28.3	0.063	3.1
33	0.80	52.9	14.9	38.7	93.1	0.138	7.8	0.066	7.1
34	0.41	58.4	12.5	56.3	71.6	0.203	14.4	0.085	7.4
35	0.65	41.3	12.1	40.8	64.5	0.121	13.2	0.076	5.7
36	1.03	236.0	16.7	45.7	87.5	0.076	29.3	0.106	6.9
37	0.49	26.7	8.1	30.4	48.6	0.052	14.3	0.089	10.8
38	1.04	64.7	8.4	30.6	53.7	0.084	12.9	0.075	4.3
39	0.49	14.2	8.2	26.4	44.8	0.069	6.9	0.082	5.5
40	0.79	77.7	13.8	37.4	76.1	0.112	11.6	0.100	7.2
41	0.78	96.9	12.6	34.7	63.7	0.063	25.6	0.083	10.7
42	0.49	21.3	7.4	25.0	37.9	0.044	15.3	0.082	8.4
43	0.54	18.3	5.3	12.3	33.3	0.032	5.9	0.028	3.2
